# COVID-19 Prevention and Mitigation Decision-Making Processes While Navigating Chronic Disease Care: Perspectives of Black Adults with Heart Failure and Diabetes

**DOI:** 10.1007/s40615-023-01862-5

**Published:** 2024-05-03

**Authors:** Leslie C. M. Johnson, Robina Josiah Willock, Sierra Simmons, Sarahna Moyd, Demetrius Geiger, Jalal K. Ghali, Rakale C. Quarells

**Affiliations:** 1 Department of Family and Preventive Medicine, Emory University School of Medicine, 1518 Clifton Rd, Atlanta, GA USA; 2 Morehouse School of Medicine, 720 Westview Drive, Atlanta, GA USA; 3 Biology Department, Spelman College, 350 Spelman Lane SW, Atlanta, GA USA; 4 Gangarosa Department of Environmental Health, Rollins School of Public Health, 1518 Clifton Rd NE, Atlanta, GA USA; 5 Health Equity Programs Department, CHC: Creating Healthier Communities, 1199 North Fairfax Street, Alexandria, VA USA

**Keywords:** Heart failure, Diabetes, COVID-19, Trusted sources

## Abstract

**Background:**

Heart failure and diabetes are comorbidities that disproportionately contribute to high morbidity and mortality among Blacks. Further compounding the racial and ethnic disparities in COVID-19 health outcomes, Blacks with cardiometabolic diseases are at high risk of experiencing serious complications or mortality from COVID-19. This study aimed to assess how Blacks with heart failure and diabetes navigated chronic care management during the COVID-19 pandemic.

**Methods:**

A mixed methods study including in-depth interviews and surveys with adults diagnosed with heart failure and diabetes (*n* = 17) was conducted in 2021–2022. Verbatim transcripts were analyzed using a thematic analysis approach.

**Results:**

Participants reported that while the pandemic initially caused delays in access to health services, shifts to telemedicine allowed for continued care despite preferences for in-person appointments. Various sources of information were used in different ways to make decisions on how to best reduce health risks due to COVID-19, but individuals and institutions affiliated with science and medicine, or who promoted information from these sources, were considered to be the most trusted sources of information among those who relied on outside guidance when making health-related decisions. Individuals’ self-awareness of their own high-risk status and perceived control over their exposure levels to the virus informed what COVID-19 prevention and mitigation strategies people used.

**Conclusion:**

Information backed by scientific data was an important health communication tool that alongside other factors, such as fear of mortality due to COVID-19, encouraged individuals to get vaccinated and adopt other COVID-19 prevention and mitigation behaviors.

## Introduction

Heart failure (HF) is a chronic, debilitating, and usually progressive disease that disproportionately affects Blacks [1, 2]. Of the 6.5 million Americans with HF, about 20% die within 1 year after diagnosis, and 50% die within 5 years of diagnosis [3, 4]. Comorbidities such as diabetes contribute to the increased hospitalizations, morbidity, and mortality of persons with HF [5, 6], with the prevalence of diabetes in this population ranging from 10 to 47% (21–28) [7]. Self-management and timely care are key to co-managing these conditions and emerging evidence suggests that the management of diabetes reduces clinical events in patients with HF [8]. Health systems faced challenges in meeting these patients’ needs even before the emergence of novel coronavirus, i.e., severe acute respiratory syndrome-coronavirus-2 (SARS-CoV-2), or COVID-19, with evidence that racial and ethnic minority groups experienced greater barriers to quality care [9–11].

The COVID-19 pandemic has only drawn further attention to the health disparities experienced by people who have been diagnosed with comorbid chronic conditions, such as HF and diabetes. It has been well documented that people with cardiometabolic diseases have a greater risk of serious complications or mortality from COVID-19, compared to people without these comorbidities [12–14]. And similar to the chronic conditions of HF and diabetes, COVID-19 disproportionately affects racial and ethnic minority groups and vulnerable populations, such as people living in poverty or with poor access to care [15].

With the recognition of these high-risk patient populations, plans to minimize COVID-19 exposure and maintain timely care to persons with HF and diabetes needed to be prioritized. Existing studies have documented how people with HF experienced delays in care during the initial phase of the COVID-19 pandemic [16], likely the result of patients’ fears of contracting the virus at health facilities [17]. These same trends occurred in Georgia, with findings that minority racial groups were disproportionately affected [18]. Promoting the use of COVID-19 prevention approaches in this population was therefore critical. Studies to date have found that people with pre-existing conditions perceive themselves to be at a higher risk of becoming ill from COVID-19 [19, 20], with perceived risk being correlated with preventive behaviors [21, 22]. Qualitative research to examine how vulnerability to COVID-19 impacted engagement in chronic disease care and self-management among Black populations affected by chronic diseases is needed. However, few studies have sought to understand how social determinants such as race/ethnicity and geography contribute to, and differentially shape, individuals’ perception of risk of contracting COVID-19 [23, 24].

In order to address persistent racial disparities in chronic disease outcomes, there is a need to understand how Blacks with HF and diabetes navigated chronic care self-management during the COVID-19 pandemic. These data could help to improve health services to this population, particularly during times of crises that may disrupt healthcare delivery. In this paper, we elicited people’s lived experiences and perceptions of chronic disease self-management during the COVID-19 pandemic with a twofold aim of understanding how care for diabetes and HF was impacted and determining the impact of individuals’ motivations on practicing COVID-19 prevention and mitigation behaviors given their preexisting chronic disease diagnoses. Findings from this study will provide nuanced insight into ways to prevent the spread and severity of COVID-19 in vulnerable populations by identifying culturally appropriate ways to engage community members in COVID-19 prevention and testing.

## Methods

### Design

We conducted a sequential mixed methods study exploring perceptions of risk to COVID-19 and experiences of accessing care during the COVID-19 pandemic among Blacks with HF and diabetes. This paper reports findings from the 17 semi-structure participant interviews conducted, alongside survey data characterizing individuals based on their socio-demographic background and self-reported data on trusted sources of information and utilization of COVID-19 prevention and mitigation practices, including vaccination.

### Participant Recruitment

Ethnic and cultural differences in health beliefs and behaviors shape the interpersonal response to disease and illness. Therefore, the experiences of our team in conducting community-engaged research in the areas of HF and diabetes among underserved minority populations were critical to our endeavors to connect with and understand the impact of COVID-19 on this population. This project leveraged relationships with community members and Federally Qualified Health Centers (FQHCs) operating across Georgia to identify and engage participants from metro Atlanta and Albany. A maximum variation sampling approach was used to recruit people with HF and diabetes who varied on key characteristics, including sex, age, and education level.

Interested individuals contacted the study team and completed a screening call to determine eligibility. Inclusion criteria were as follows: (1) ≥ 18 years, (2) self-identified as Black or African American, (3) English-speaking, and (4) diagnosis of HF and diabetes. Individuals determined to be eligible were invited to participate in an interviewer-administered survey and an in-depth interview, to be completed in sequence. Data collection took place after enrolled participants provided informed consent. This study was approved by the Morehouse School of Medicine institutional review board.

### Data Collection

We collected data over the phone between May 2021 and June 2022, and all data collection activities were conducted by research staff self-identified as African American or Black.

Participants first completed a survey consisting of several validated measures used to collect information on HF and diabetes self-care behaviors, psychosocial health, along with basic demographic information and questions including COVID-19 behaviors and experiences (e.g., sheltering in place), access to and use of social media, and trusted sources of information. To assess access and use of social media, participants were asked “Do you have a smart device (tablet or smart phone)?” and how they have used the internet in the past 12 months to engage with other people online through social networks (e.g., Facebook). To assess trust in information sources, participants responded to Likert type items on the degree to which they trust each named source (i.e., healthcare provider, religious leader, close friends or family, coworkers/classmates/acquaintances, news/mass media, social media, US government, US Coronavirus Task Force) to provide correct information about COVID-19. Likert scale response options included, “not at all,” “a little,” “a great deal,” and “don’t know.” Study data were collected and managed using REDCap (Research Electronic Data Capture) electronic data capture tools hosted at Morehouse School of Medicine [25]. REDCap is a secure, web-based application designed to support data capture for research studies, providing: (1) an intuitive interface for validated data entry; (2) audit trails for tracking data manipulation and export procedures; (3) automated export procedures for seamless data downloads to common statistical packages; and (4) procedures for importing data from external sources. The interviewer-administered survey lasted approximately 60 min for each participant. Participants were then scheduled for an in-depth interview. Interviews lasted between 50 and 70 min.

The interview guide was developed and critically revised by the full study team. The guide was pilot tested and revised prior to data collection to ensure all open-ended questions and probes elicited sufficient context regarding people’s beliefs, attitudes, and preventative behaviors regarding COVID-19. The interview guide topics included: knowledge and perceptions of COVID-19, perceptions of risk and susceptibility to COVID-19, the impact of COVID-19 on access to care and self-management, COVID-19 prevention and mitigation strategies, and the impact of COVID-19 on social isolation, loneliness, and mental health. All study team staff responsible for conducting data collection received training in interview techniques and had an opportunity to take notes during an interview conducted by a senior qualitative interviewer prior to conducting an interview themselves. Random transcript checks were completed throughout active data collection to determine data quality and provide interviewers with feedback on how to effectively probe participant responses. The guide was iteratively revised to include emergent topics.

### Data Analysis

Descriptive analyses were performed to determine the distribution of key sociodemographic variables using mean with standard deviation (SD) for numerical variables, and frequency with percentage for categorical variables. Survey data were used to probe qualitative interview responses.

Following completion of the interviews, audio-recordings were transcribed and checked for accuracy. Names and other identifying information of participants were removed. To develop the initial codebook, two research staff members independently reviewed three transcripts. They met to discuss and finalize the codebook, which was iteratively updated to include emergent codes, such as “spirituality” and “social influences and COVID”, while coding the remaining transcripts. Throughout the coding process the study team met periodically to discuss discrepancies in coding and come to a team consensus on final code applications. Coding was completed using Dedoose, a cross-platform web-based application designed to analyze qualitative and mixed methods research with text, photos, and audio files [26]. Thematic analysis was used to identify the range and prioritization of salient issues related to how COVID-19 impacted access to care, diabetes and HF management, and risk for adverse health outcomes. Data were compared based on participant characteristics (e.g., sex, geographic location), COVID-19 prevention behaviors, and what their trusted sources of information were. Additional interviews were conducted until no new information was felt to be contributed from the interviews (i.e., saturation was achieved) using purposive sampling based on race and diagnoses of HF and diabetes.

## Results

Seventeen participants completed the project’s qualitative interview and provided complete survey data (see Tables 1 and 2). In keeping with the goals of this project, all participants were Blacks. The average age of participants was 58 years and there was near gender parity, with 9 men (52.9%) and 8 women (47.1%). At the time of the interview, the majority of participants lived alone (70.6%) and were either divorced/separated or widowed (52.9%), around 70% were unemployed or unable to work, approximately 65% had at least a high school education, and 71% had household income less than $25,000. Most participants reported having access to a smart device (70.6%), such as a tablet or smart phone, and nearly half of participants reported using the internet to visit social networking sites (47.1%). Participants rated their health status as fair and poor at 53% and 35%, respectively. Only one participant rated their health as excellent. All 17 participants reported receiving at least one dose of the Pfizer, Moderna, or Johnson and Johnson COVID-19 vaccine. Further, 29% reported receiving two doses of the Moderna vaccine, 47% reported receiving two doses the Pfizer vaccine, and 17% reported receiving the single-dose Johnson and Johnson COVID-19 vaccine.

**Table 1:** Participant characteristics

Characteristic	*N* (%) or mean (SD)
Race/ethnicity^a^	
Black/African American	17 (100)
Hispanic ethnicity	0
Other ethnicity	1 (5.9)
Gender	
Man	9 (52.9)
Woman	8 (47.1)
Age	58 (7.1)
Relationship status	
Married/unmarried couple	2 (11.8)
Divorced/separated	6 (35.3)
Never married	6 (35.3)
Single	0
Widowed	3 (17.6)
Education completed	
Less than/some high school	6 (35.3)
High school graduate	2 (11.8)
Some college	6 (35.3)
Associate’s degree	2 (11.8)
Bachelor’s degree	1 (5.9)
Employment status	
Employed	3 (17.6)
Unemployed	3 (17.6)
Retired	2 (11.8)
Not able to work because of a disability	9 (52.9)
Pretax family income	
< 15 k	8 (47.1)
15–24,999 k	4 (23.5)
25–49,999 k	1 (5.9)
50–99,999 k	1 (5.9)
> 100 k	1 (5.9)
Prefer not to answer	2 (11.8)
Household size	
1	12 (70.6)
2	2 (11.8)
3–5	2 (11.8)
≥ 6	1 (5.9)
Self-rated health	
Excellent	1 (5.9)
Fair	9 (52.9)
Poor	6 (35.3)
Missing	1 (5.9)
Access to smart device	12 (70.6)
Purpose of internet use ^a^	
Visit social networking sites	8 (47.1)
Share health information on social networking sites	1 (5.9)
Write in an online diary or blog	3 17.6)
Participate in an online forum or support group for people with a similar health or medical issue	2 (11.8)
Watch a health-related video on YouTube	6 (35.3)

^a^Percentages do not add up to 100% as respondents could select more than one response option

**Table 2:** Trusted sources of COVID-19–related health information

Information sources	Level of trust (*N* = 17)			
	Not at all (%)	A little (%)	A great deal (%)	Don’t know (%)
Healthcare provider			17 (100)	
Faith leader	1 (5)	2 (11.8)	14 (82.4)	
Friends/family	4 (23.5)	3 (17.6)	10 (58.8)	
Coworkers/classmates/acquaintances	9 (52.9)	4 (23.5)	4 (23.5)	
News/mass media	3 (17.6)	6 (35.3)	6 (35.3)	2 (11.8)
Social media	9 (52.9)	3 (17.6)	1 (5)	4 (23.5)
US Government	6 (35.3)	6 (35.3)	4 (23.5)	1 (5)
US Coronavirus Taskforce	2 (11.8)	6 (35.3)	7 (41.2)	2 (11.8)

The pandemic also brought the individuals and entities that are considered as trusted sources of health-related information into sharp relief (see Table 2). We found high levels of trust among participants for traditionally highly trusted sources of health information like healthcare providers, with 100% of participants reporting that they trusted their healthcare providers “a great deal”. Faith leaders and friends/family were also very trustworthy, with 82% of respondents saying they trusted their faith leaders, and 58% their friends/family “a great deal.” There was consistent skepticism for social media, classmates/coworkers, and acquaintances as trustworthy source of information. These sources earned no trust “at all” among more than 50% of respondents. Further, more than 25% of respondents were unsure whether social media information could even be trusted. The majority of respondents indicated that they trusted the Coronavirus Task Force either “a little” (35.3%) or “a great deal” (41.2%). Similarly, respondents indicated that they trusted the US government either “a little” (35.3%) or “a great deal” (23.5%).

Three themes were generated from the interview data and collectively describe how COVID-19 impacted chronic disease care during the pandemic, the ways in which COVID-19 information was perceived based on the disseminating source, and how that information was then used to determine behaviors for reducing exposure to the virus and for monitoring risk on an on-going basis.

### Theme 1: Maintenance of Chronic Disease Care During COVID-19

Nearly all persons reported that they felt COVID-19 had not impacted their access to care for diabetes and heart failure self-management. Many participants expressed that they had experienced some delays in their care, particularly as healthcare systems implemented new policies and practices, but all participants reported being able to communicate with their usual care provider during those transitional periods. As individuals described the circumstances surrounding their HF and diabetes care appointments, several mentioned that they either had not been attending regular appointments pre-COVID and refrained from making clinical appointments during the initial phases of the pandemic, while the remainder reported that they still attended appointments either in-person, by phone, or zoom. Regardless of their patterns of healthcare utilization pre-COVID, participants shared the sentiment of one individual who stated: “I still got the care that I needed, but it was just different.” One male living in the city elaborated that, “It just kind of slowed it down for a minute, but it changed. Now I have a new schedule and routine, so it’s sort of getting back to normal maybe.” Similarly, a woman living in a rural community reflected:

You know the first year [of the pandemic] it changed because I didn’t go to the doctor as much. But since I started going back to the doctor…we kind of just keep it checked…so, it really hadn’t—COVID didn’t really affect my diabetes.

Those who went to the clinic reported that the primary difference was that there were numerous COVID-19 prevention policies in place (e.g., social distancing in the waiting room or waiting in your vehicle, taking temperatures at the door, masking requirements). Though several people mentioned that initially they did not maintain a diet and physical activity level that supported their chronic disease self-management, most people shared that after the first year of the pandemic they had returned to their routines and many reported checking their glucose levels on a more frequent basis.

There were conflicting views on the acceptability of telemedicine options among individuals who engaged with the healthcare system while COVID-19 prevention policies were in place. One individual best summed up the attitude of those that accepted the switch to telehealth, stating, “My cardiologist called me–she said, I just want you to know that I’m going to be available to you for anything. We're going to be doing online doctor appointments…that has been the case up until now. So no problems.” Individuals who were not in favor of these appointments described them as “informal,” “not personal enough,” and a format that negatively impacted their ability to communicate with their physician. In regard to phone appointments, individuals did not like that they could not see their physician or know that they were referencing their medical chart when they had a consultation. Additionally, many participants felt that the expense of the telehealth appointment was too high given that the physician could not do a physical exam. Participants felt that the quality of care was better when appointments were in-person and that they always chose to go in-person if that option was provided.

### Theme 2: Discerning Trusted Sources of Health Information to Guide Health Behavior During the COVID-19 Pandemic

All participants were asked what sources they considered trustworthy or untrustworthy with regard to providing correct information about COVID-19. Though individuals reported seeking information about COVID-19 from a range of sources, there were three types of ways that people described their engagement with trusted and distrusted information on COVID-19 during the pandemic.

The first group, information purists, had a clearly defined group of information sources that they trusted to get COVID-19 and other health information from, and they reported ignoring information from entities outside of those sources. The second group, information sifters, also had pre-identified trusted sources, but described how they would seek out additional information from untrusted sources to gain a balanced understanding of the public debate on a topic (e.g., utility of masking to prevent the spread of COVID-19). For instance, one person who explicitly named Fox News a distrusting source, described how they continued to watch that channel to maintain a balanced perspective, stating, “I try to be equal and listen to both, you know CNN, MSNBC, and Fox to get a clearer view to make a decision.” People in this group expressed how seeing agreement across multiple sources made them more confident in the quality of the information they were receiving on COVID-19. The named trusted sources were the same across both groups, with healthcare providers being named as a trusted source by nearly all participants, followed by governmental health agencies (e.g., the Centers for Disease Control and Prevention [CDC]), scientists, and close friends or family who have a degree, training, or job in medicine, public health, or an allied health profession. When asked why scientists were their most trusted source of health information, one individual elaborated, “They study the different viruses for a living. They’re the ones who make up the vaccines to take care of you. I would trust them better than I would say, for instance, a [pharmaceutical company] like Moderna or Pfizer.” Several people also named religious leaders as trusted sources, but only under the circumstances through which those leaders brought in external community health experts and providers (e.g., hosted health fairs with COVID-19 vaccination and testing; invited health department speakers). Similarly, across both of these groups, all but one person reported distrusting television news and social media as sources of information on COVID-19. The third group, the information agnostic, reported not fully trusting any source of information despite watching the news and taking in information from multiple sources. As one woman stated:

There was one outlet that I trusted more than another, and I didn’t 100% trust any of them. That’s why I would speak to my doctor or if my son had to go to the doctor, I would talk to his doctor about it. And I would also research it myself ‘cause I really have a hard time believing anything from anybody. So, I just have to take everything in and decided for myself what I want to do.

Compared to individuals in the other two groups, these individuals had lower levels of education and income.

### Theme 3: Strategies for Reducing Risks Related to COVID-19

All participants in the study believed that they were at a higher risk of dying if they contracted COVID-19 due to factors related to their race, age, and health status. Media attention covering high mortality in the Black community as a result of the pandemic made many participants fearful of contracting the virus, especially when some experienced loss within their own social circles. Other named vulnerabilities included advanced age, being overweight, and having an underlying health condition such as heart failure or diabetes. These factors, alongside re-enforcing guidance from trusted sources (i.e., healthcare providers and published scientific data) for many, and led everyone to get vaccinated. Additionally, just over half of individuals felt they were also at a higher risk of exposure to COVID-19 because they perceived themselves to be unable to exert control over aspects of their life that introduced exposure to the virus, such as sharing a household space with children or essential workers and being dependent on public transportation.

**Fig. 1:**
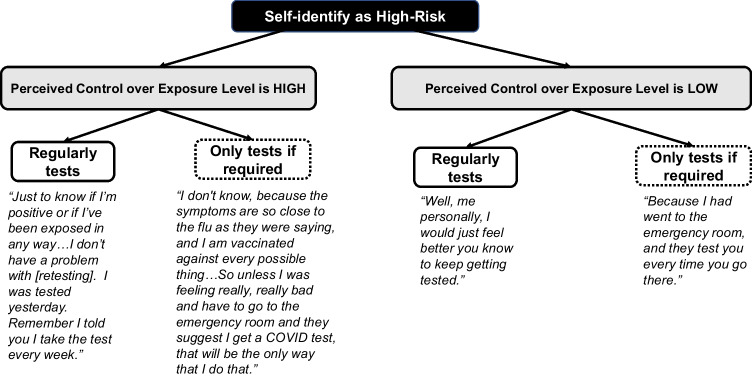
Differences in COVID-19 prevention and mitigation behaviors

COVID-19 testing behaviors differed according to how much control participants felt they had over their exposure to the virus (see Fig. 1). Self-perception of high versus low control identified different sets of circumstances that led to getting regular COVID-19 tests or only being tested if required to by a policy they had to abide by (e.g., hospital testing policy). Individuals with high perceived control over exposures to COVID-19 typically lived alone, were unemployed or retired, and strictly enforced visitor policies in their home that limited contact with people outside their home. On the other hand, individuals with low perceived control over exposures to COVID-19 often lived in a shared household or apartment complex, were employed, or had interactions with family members who did not chose to vaccinate or wear masks.

Among the group of individuals with high perceived control over exposures to COVID-19, there was less of a perceived need for routine COVID-19 testing because these individuals were better able to self-isolate and therefore did not have a need to test. Those who did test regularly expressed concerns over potential exposures to emergent COVID-19 variants and the high death rates from COVID-19 in the Black community. Individuals in both groups reported wearing masks and using hand sanitizer to prevent exposure to COVID-19 whenever they had to leave their home.

Among the group of individuals with low perceived control over exposures to COVID-19, the majority chose to get regularly tested for COVID-19 because their work or living situation forced them to occupy crowded spaces that increased their likelihood of exposure. Individuals in both groups described being aware that they had risk factors (e.g., advanced age, underlying health condition, being a racial minority) that made them more likely to experience adverse health outcomes if they were to get infected, which led everyone to adopt multiple COVID-19 prevention strategies (e.g., vaccination, masking, hand washing, social distancing). But only those who felt they could adequately self-isolate within their home chose not to seek out COVID-19 testing. People with shared living spaces who did not try to self-isolate instead developed cleaning rituals for re-entering their home that they felt would keep anyone entering the house from spreading the virus.

Anyone who reported getting tested regularly for COVID-19 viewed testing as a way to have reassurance and actionable information from which to make decisions about their health. Individuals who did not test unless required reported having been tested for COVID-19 when testing first became available, largely because it was convenient and free, but they did not feel it was necessary after they were vaccinated.

## Discussion

This study assessed the extent to which Black adults with heart failure and diabetes felt that their medical care had been impacted by the COVID-19 pandemic and the ways in which they responded to minimize the potential for adverse health outcomes as a result of COVID-19. The results showed that people adjusted to shifts in the medical system when seeking care for their HF or diabetes, although, they preferred to have in-person interactions with their medical providers. The most commonly reported sources for information about COVID-19 included television media, healthcare providers, and CDC guidance. Some participants who indicated in their survey responses that they trusted family, friends, or people in their social network to provide correct information about COVID-19, clarified during interviews that this was only the case when those individuals had education, training, or worked within an organization that could lend credible insight on COVID-related matters (e.g., having a relative that works at the CDC). Similarly, during interviews, several individuals clarified that they trusted religious leaders when they brought in external health experts and practitioners to provide guidance and health services. While most people took in a variety of news sources, only a subset of individuals factored in the perspectives of informational sources they did not trust when seeking out and assessing the quality of information available on COVID-19. Despite this, people aligned themselves with sources affiliated with science and medicine when making health-related decisions. Further, a few individuals indicated in their survey responses that they trusted healthcare providers a great deal yet reported in their interview that they do not fully trust any source of information and instead rely on their own judgment when making decisions about their health. Among the three information typologies identified in this study, the differences in lived experiences among participants based on socio-economic status may have attributed to why the information agnostic distrusted all sources of information; this group had lower reported levels of education and income than information purists and shifters.

In this study, all participants reported being vaccinated against COVID-19. As of July 2022, however, Black people represent the smallest racial/ethnic group in the USA to have received at least one COVID-19 vaccine dose [27]. Findings from this study indicate that people received two consistent health messages from media coverage during the early pandemic that led them to get vaccinated. First, media coverage regarding racial/ethnic disparities in COVID-19 related deaths re-enforced what people experienced within their own social circles. Second, news coverage of data demonstrating that people with underlying chronic conditions are at an increased risk for health complications invoked concerns for how individuals could best protect themselves from exposure to the virus. While others have advocated for coordinated public health messaging to relay accurate information about critical health risks during times such as a pandemic [28], these findings demonstrate that pervasiveness and frequency of a public health message across different information sources may also function to inform people’s health risk perceptions.

Individuals’ self-awareness of their own high-risk status and perceived control over their exposure to the virus informed what COVID-19 prevention and mitigation strategies people used. Our results suggest that individuals were motivated to adopt multiple forms of COVID-19 prevention and mitigation behaviors based on factors related to their identity, health status, physical and social environment, as well as the information they received about COVID-19 from trusted information sources. These findings mirror those of Bateman and colleagues, who identified concerns about contracting COVID-19 and clear and consistent messages from trusted sources as factors that facilitated Blacks in under-resourced communities to adopt COVID-19 prevention strategies [24]. Furthermore, while an international study across ten countries found that sources of information are generally shared across countries, they documented variability across cultures regarding risk perception and the adoption of preventative health behaviors [22]. Other studies have found racial and ethnic differences in COVID-19 testing behaviors to be associated with differences in testing motivation [29]. Our findings expand upon these studies by detailing processes by which people from a vulnerable group discern and engage with information sources, and how they then use the information they receive to make decisions regarding their health behavior (e.g., getting regular COVID-19 tests). Bateman et al. also found that individuals who experienced difficulty in social distancing had a more challenging time adhering to prevention methods [24], which was critical in determining the types of COVID-19 prevention and mitigation strategies people with chronic conditions chose to utilize in our study. The most notable contrast, however, was that our study participants were keenly aware of their health risks and how COVID-19 was impacting the Black community. Because our study was conducted further into the pandemic, participants in our study had more time to be exposed to news media and other sources reporting on COVID-19. Future research is needed to better understand how perceptions of risk for vulnerable groups changes over time.

This study was strengthened by the purposive sampling of individuals from diverse socio-economic backgrounds and rural communities. Due to COVID-19, all interviews took place over via Zoom. This may have impacted the quality of the interview, but steps were taken to build rapport with participants including having the same person administer the survey and conduct the interview when possible. Additionally, because all the recruited participants reported having received the COVID-19 vaccine, we were unable to compare how behavioral motivations and access to care may have been different among individuals who were unvaccinated. Future studies should seek to purposively sample based on this characteristic in order to represent the perspectives of this sub-group.

## Conclusion

Given the persistent racial disparities in HF, diabetes, and COVID-19 health outcomes, it is important to understand the perceptions of Blacks with chronic disease and report their experience. This information provides a valuable contribution to public health efforts aimed at minimizing health disparities. Public health messaging should be disseminated across trusted information sources in parallel with community-based resources that promote the uptake of health promoting behaviors. With an understanding of the diverse motivations that racial minority groups have for adopting COVID-19 prevention and mitigation behaviors, we can design effective strategies to reduce the risk of virus transmission among vulnerable groups, such as people with underlying chronic diseases.

## Data Availability

The data that support the findings of this study are available on request from the corresponding author, RQ. The data are not publicly available due to their containing information that could compromise the privacy of research participants
